# Molecular Characteristics of Choledochal Cysts in Children: Transcriptome Sequencing

**DOI:** 10.3389/fgene.2021.709340

**Published:** 2021-08-03

**Authors:** Yong Lv, Xiaolong Xie, Lihui Pu, Qi Wang, Jiayin Yang, Siyu Pu, Chengbo Ai, Yi Liu, Jing Chen, Bo Xiang

**Affiliations:** ^1^Laboratory of Pediatric Surgery, Department of Pediatric Surgery, West China Hospital, Sichuan University, Chengdu, China; ^2^Department of Critical Care, West China Hospital, Sichuan University, Chengdu, China; ^3^Liver Transplant Center, Department of General Surgery, West China Hospital, Sichuan University, Chengdu, China; ^4^Department of Rheumatology and Immunology, Rare Diseases Center, Institute of Immunology and Inflammation, Frontiers Science Center for Disease-Related Molecular Network, West China Hospital, Sichuan University, Chengdu, China

**Keywords:** transcriptome, choledochal cysts, weighted gene co-expression network analysis, children, bioinformatics

## Abstract

A choledochal cyst (CC) is a common congenital biliary disease in children, yet the underlying molecular bases for the cystic and fusiform clinical subtypes are unknown. RNA sequencing (RNA-seq) has been performed on 22 high-quality CC samples, including 12 cystic CC and 10 fusiform CC samples, to search for molecular features. Weighted gene co-expression network analysis (WGCNA) was performed to identify key modules associated with clinical subtypes. Bioinformatic analyses were conducted to elucidate potential mechanisms. Then, we constructed protein–protein interaction (PPI) networks to identify candidate hub genes related to CC. Finally, we used the support vector machine (SVM) to eliminate redundant features and screen out the hub genes. The selected gene expression was determined in CC patients through quantitative real-time polymerase chain reaction (PCR). A total of 6,463 genes were found to be aberrantly expressed between cystic CC and fusiform CC. Twelve co-expression modules that correlated with clinical subtypes of CC were identified and assigned representative colors. Among the 12 modules, the blue module was considered the key module. Two functionally distinct sets of dysregulated genes have been identified in two major subtypes, metabolism-related genes in cystic CC and immune-related genes in fusiform CC. A total of 20 candidate hub genes that were correlated with clinical subtypes were found in the blue module. In addition, we found ERBB2 and WNT11 that have not been studied in CC and verified their differential expression in CC through quantitative real-time PCR experiments. For the first time, we have described the transcriptome characteristics of CC. These results suggest that cystic CC and fusiform CC have different molecular mechanisms. The bi-omics-identified novel candidate genes and pathways might be helpful for personalized treatment and are of great clinical significance for CC.

## Introduction

A choledochal cyst (CC), also known as congenital biliary dilatation (CBD), is a common congenital biliary disease in children characterized by dilatation of extrahepatic biliary tract. The incidence of CCs in Asians is significantly higher than that in Europeans and Americans, and the incidence in Asian populations can be as high as 1/1,000 (van den Eijnden et al., 2017). CCs mostly occur in infant and children, particularly in females, and the incidence ratio of male to female is about 1:4 (Atkinson and Davenport, 2014). The etiology of CC is unknown, which may be related to an anomalous pancreaticobiliary junction, bile duct dysplasia, and other factors (Friedmacher et al., 2019). However, these theories cannot explain the pathogenesis of all types of CCs. There are obvious differences in epidemiology, imaging, and clinical features among different types of CCs. CCs were divided into five types in 1977 by Todani et al. (1977). The classification was complicated and inaccurate. Todani’s type II and type III pathologies were rare in children, and type V was Caroli disease that requires a different management. It fails to provide a surgical guideline specific to the subtypes (Makin and Davenport, 2012). The main subtypes of CCs are cystic CCs (Ia) and fusiform CCs (Ic). At present, dividing CCs into two types (cystic and fusiform extrahepatic dilatation) is the most widespread classification method, which has great advantages in clinical diagnosis and treatment (Diao et al., 2011).

The two principal extrahepatic phenotypes, cystic (Ia) and fusiform (Ic) extrahepatic dilatation, making up more than 95% of CCs, have clear clinical and physiological differences. Different subtypes require different operative strategies (Jung et al., 2012). These types of CCs were closely related to malignant transformation (Soares et al., 2014). However, the molecular processes associated with CCs remain largely unknown, and there are insufficient molecular criteria to distinguish clinical subtypes. Some CC cases occur in families, suggesting that genetic predisposition plays a role in its pathogenesis (Clifton et al., 2006). Patients with cystic biliary dilatation have more severe hepatic dysfunction, and the ages were younger than those with fusiform biliary dilatation. We do not know, however, whether cystic biliary dilatation and fusiform biliary dilatation have a distinct pathogenesis at the molecular level. No microarray studies and RNA sequencing (RNA-seq) were used to identify distinct molecular processes for CCs. So far, further basic science and research into the etiology of CCs were absent, whether the molecular pathways are different in clinical subtypes of this complex disorder. In addition, if the transcriptomes of homogeneous samples between the cystic and clostridial subtypes are compared, a more complete set of dysregulated genes in each subtype may be found.

Weighted gene co-expression network analysis (WGCNA) is a systems biology approach to describe gene association patterns between different samples (Liu et al., 2021). According to the association between gene sets and phenotypes, it can be used to identify highly collaborative gene sets and to identify candidate biomarker genes or therapeutic targets (Panahi and Hejazi, 2021). Machine learning is one of the important branches of artificial intelligence science, which can mine new data features and results from massive data. With the development of high-throughput sequencing technology, more genomics data need to be processed, and the application of machine learning can solve this problem (Panahi et al., 2019). The broad goal of this study was to systematically explore CC molecular signatures associated with clinical subtypes. We carried out transcriptome sequencing on high-quality CC samples. Furthermore, we used the WGCNA to find highly correlated gene modules with clinical traits and to identify the key genes in selected modules. The discovery of novel candidate CC-related gene is important for subsequent functional and mechanistic studies of CCs.

## Materials and Methods

### Study Participants

The study was approved by the West China Hospital of Sichuan University Biomedical Research Ethics Committee, and informed consent was obtained from all participants. Twenty-two patients with CCs (12 cystic and 10 fusiform extrahepatic dilatation) were consecutively recruited between January and December 2020 from the Pediatric Surgery Department of West China Hospital, Sichuan University. All children underwent a collection of medical history and had hepatobiliary ultrasonography and magnetic resonance cholangiopancreatography. All children underwent cyst excision and Roux-Y hepaticojejunostomy. The CC samples were collected from all participants and stored in liquid nitrogen. Transcriptomics analysis was performed by Novogene Co., Ltd. (Beijing, China). The raw data in this article have been deposited in National Center for Biotechnology Information’s (NCBI) Gene Expression Omnibus and are accessible through GEO Series accession number GSE179075^1^.

### Differentially Expressed Gene Screening

Differentially expressed genes (DEGs) between cystic and fusiform CCs were screened from the RNA-seq data with the “edgeR” package. Significantly changed genes were selected with *p*-value < 0.05 and log_2_| fold change| ≥ 1.

### Construction of Weighted Gene Co-expression Network

The co-expression network of the DEGs was constructed by the R package “WGCNA” (Langfelder and Horvath, 2008). The network construction procedure included the following main steps: (1) select the appropriate soft threshold (weighting coefficient) and define the similarity matrix; (2) the similarity matrix was then converted into an adjacency matrix by using a power adjacency function; (3) transform the adjacency matrix into a topological overlap matrix (TOM); (4) the hierarchical clustering was used to produce hierarchical cluster tree from TOM-based dissimilarity (dissTOM); and (5) modules were defined as branches of the hierarchical cluster tree using the dynamic tree cut method.

### Identification of Clinically Significant Modules

The main purpose of WGCNA is to link key genes in the co-expression network with external clinical traits. The association between the gene modules and clinical characteristic was analyzed as follows: (1) calculate the module eigengene (ME) of each module, and ME represents the overall expression level of the module. (2) Identify module membership (MM), and MM represented the correlation of gene expression profile with the ME. The larger the absolute value of MM, the more important the gene is in the module. (3) Extract gene significance (GS) and module significance (MS). MS is the average absolute GS measure for all genes in a given module. The higher the absolute value of GS, the more biologically significant is the gene (Langfelder and Horvath, 2008).

### Functional Enrichment Analysis

To obtain further insights into the function of the DEGs in the module most related to CCs, we referred to the Metascape^2^ to perform the enrichment analysis (Zhou et al., 2019). *p* < 0.05 was set as the cutoff. The R software was adopted to show the results graphically. Gene set enrichment analysis (GSEA) was performed to identify the enriched pathways based on the expression profiles between the cystic and fusiform CC subtypes. The R package “clusterprofiler” was utilized to conduct GSEA. The h.all.v7.2.symbols.gmt in Molecular Signatures Database (MSigDB) was selected as the reference gene set, and adjusted *p*-value < 0.05 was chosen as the cutoff criteria.

### Featured Gene Selection and the Support Vector Machine Classifier Construction

The genes selected from the WGCNA were uploaded to the STRING^3^ database, and the protein–protein interactions (PPIs) between these genes were obtained by online tools, and the network was mapped. Cluster analysis extracts tightly connected regions of biological networks and isolates modules of importance in the network. Based on the constructed PPIs, the MCODE plug-in was used to find key modules (Bandettini et al., 2012). Subsequently, selecting nodes with MCODE score ≥ 2, degree cutoff = 2, node score cutoff = 0.2, max depth = 100, and k−score = 2 and clustering analysis result in the candidate hub genes. The featured selection technique is an efficient tool for identifying meaningful information from a given gene dataset; support vector machine recursive feature elimination (SVM-RFE) is a popular feature selection technique and has exhibited promising and expanding applications for the analysis of high-dimensional data (Xu et al., 2021). We applied the machine learning method to filter candidate hub genes. The fivefold cross-validation method was used to split the data and assign random numbers. When halve above was set to 100, the order of feature vector was obtained.

### RNA Isolation and Quantitative Reverse Transcription-PCR

Another 24 CC tissues (12 cystic and 12 fusiform) were used for RNA isolation. Total RNA was isolated from CC tissue with TRIzol. To assess gene expression, 1 μg RNA was used for cDNA synthesis with NovoScript^®^, 1st Strand cDNA Synthesis SuperMix (Novoprotein Scientific Inc., China). PCR amplification was performed at 95°C for 5 min, followed by 48 cycles at 95°C for 10 s, 60°C for 10 s, and 72°C for 20 s in a real-time PCR system with SYBR green (Applied Biosystems). For each sample, we used glyceraldehyde-3-phosphate dehydrogenase (GAPDH) as inner reference to normalize target gene expression. Primers for quantitative reverse transcription-PCR (qRT-PCR) were designed with Primer-BLAST in the NCBI. Primers are listed as follows. Relative changes in gene expression were determined using the 2^–Δ^ ^Δ^ ^Ct^ method (Schmittgen and Livak, 2008).

ERBB2 forward primer: TGACACCTAGCGGAGCGAT

ERBB2 reverse primer: GGGGGATGTGTTTTCCCTCAA

WNT11 forward primer: CGATGCTCCTATGAAGGTGAAA

WNT11 reverse primer: CTTCCGTTGGATGTCTTGTTG.

## Results

### Differentially Expressed Genes Between Cystic and Fusiform CCs

**FIGURE 1:**
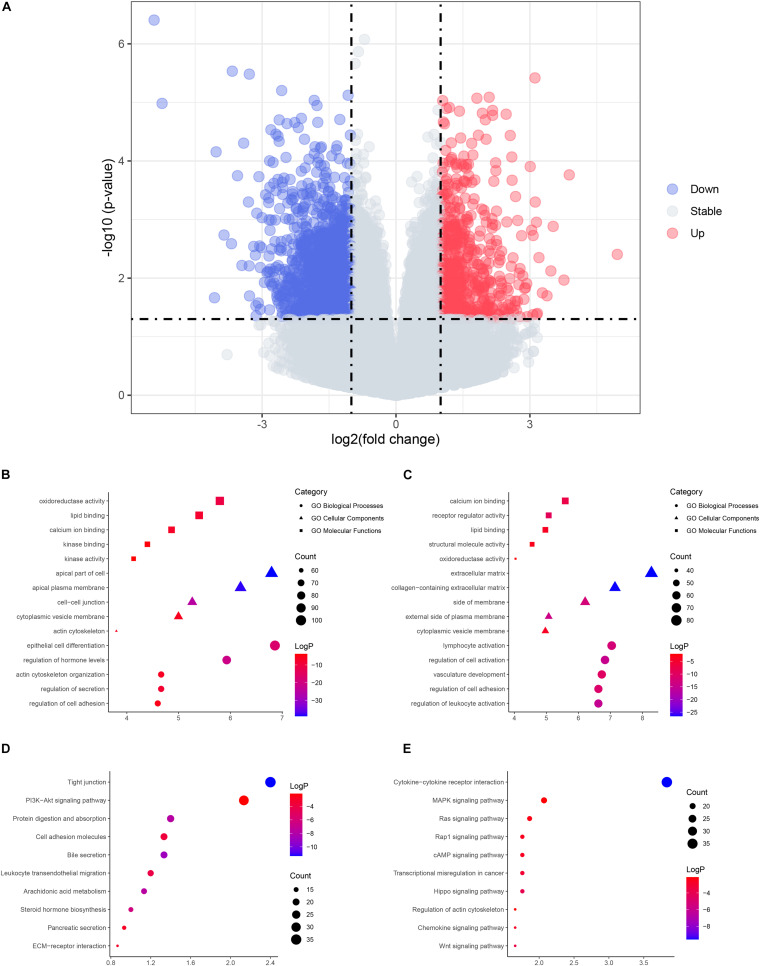
The volcano plot and enrichment analysis of differentially expressed genes (DEGs). **(A)** Volcano plot of DEGs. Red, upregulated genes in cystic choledochal cysts (CCs); blue, downregulated genes in cystic CCs. **(B)** The enriched Gene Ontology (GO) terms of upregulated DEGs. **(C)** The enriched GO terms of downregulated DEGs. **(D)** The enriched pathways of upregulated DEGs. **(E)** The enriched pathways of downregulated DEGs.

A total of 12 cystic (Ia) and 10 fusiform (Ic) CC tissues were collected in this study. The baseline characteristics of patients of each group are shown in Table 1. A total of 6,463 DEGs were identified between the two subtypes and selected for subsequent analysis. Among the DEGs, 2,750 genes were upregulated and 3,731 genes were downregulated in cystic CCs (Figure 1A).

**TABLE 1:** Baseline characteristics of the choledochal cyst (CC) patients.

	**Fusiform (Ic)**		**Cystic (Ia)**		***p*-value**
Total	10		12		
Female	5	50%	10	83.30%	0.095
Male	5	50%	2	16.70%	
Age (months)	35	(30–42)	27	(15–34)	0.069
DB (μmol/L)	12.9	(2.4–63.7)	3.8	(1.3–12.1)	0.093
TB (μmol/L)	19.9	(9.4–69.7)	9.1	(6.1–19.1)	0.140
IB (μmol/L)	5.9	(4.8–9.1)	4.8	(3.6–5.5)	0.180
ALT (U/L)	65	(15–189)	27	(16–113)	0.628
AST (U/L)	54	(36–90)	59	(33–118)	0.872
γ-GGT (U/L)	374	(84–777)	82	(40–484)	0.314
ALP (U/L)	416	(318–553)	284	(235–366)	0.140
AMY (U/L)	68	(50–71)	66	(52–166)	0.674
PT (s)	11.3	(10.6–11.9)	11	(10.8–11.1)	0.497
APTT (s)	29.3	(28.7–29.9)	28.7	(26.5–30.7)	0.418

*The parameters are presented as the median (IQR). IQR, interquartile range; DB, direct bilirubin; TB, total bilirubin; IB, indirect bilirubin; ALT, alanine aminotransferase; AST, aspartate aminotransferase; γ-GGT, γ-glutamyl transpeptidase; ALP, alkaline phosphatase; AMY, amylase; PT, prothrombin time; APTT, activated partial thromboplastin time.*

### Molecular Pathways Involved in Cystic and Fusiform CCs

Functional enrichment on these DEGs was performed to identify possible biological functions. The upregulated DEGs in cystic CCs were obviously enriched in biological processes (BPs) such as epithelial cell differentiation and regulation of hormone levels (Figure 1B). For molecular function (MF), the upregulated DEGs were enriched in oxidoreductase activity, lipid binding, and so on (Figure 1B). For cell component (CCt), the upregulated DEGs were distinctly enriched in the apical part of the cell, apical plasma membrane, and so on (Figure 1B). For BP, the downregulated DEGs in cystic CCs were obviously enriched in lymphocyte activation, regulation of cell adhesion, and so on (Figure 1C). For MF, the downregulated DEGs were enriched in calcium ion binding, receptor regulator activity, and so on (Figure 1C). For CC, the downregulated DEGs were distinctly enriched in the extracellular matrix, collagen-containing extracellular matrix, and so on (Figure 1C). The screened DEGs were analyzed by the pathway analysis for a more in-depth understanding. The upregulated DEGs were enriched in the tight junction, phosphatidylinositol-3-phosphate/AKT (PI3K/AKT)-signaling pathway, protein digestion and absorption signaling pathways, and so on (Figure 1D), while the downregulated DEGs were enriched in cytokine–cytokine receptor interaction, mitogen-activated protein kinase (MAPK) signaling pathway, Ras signaling pathway, and so on (Figure 1E).

### Gene Set Enrichment Analysis Result Between Cystic and Fusiform CCs

**FIGURE 2:**
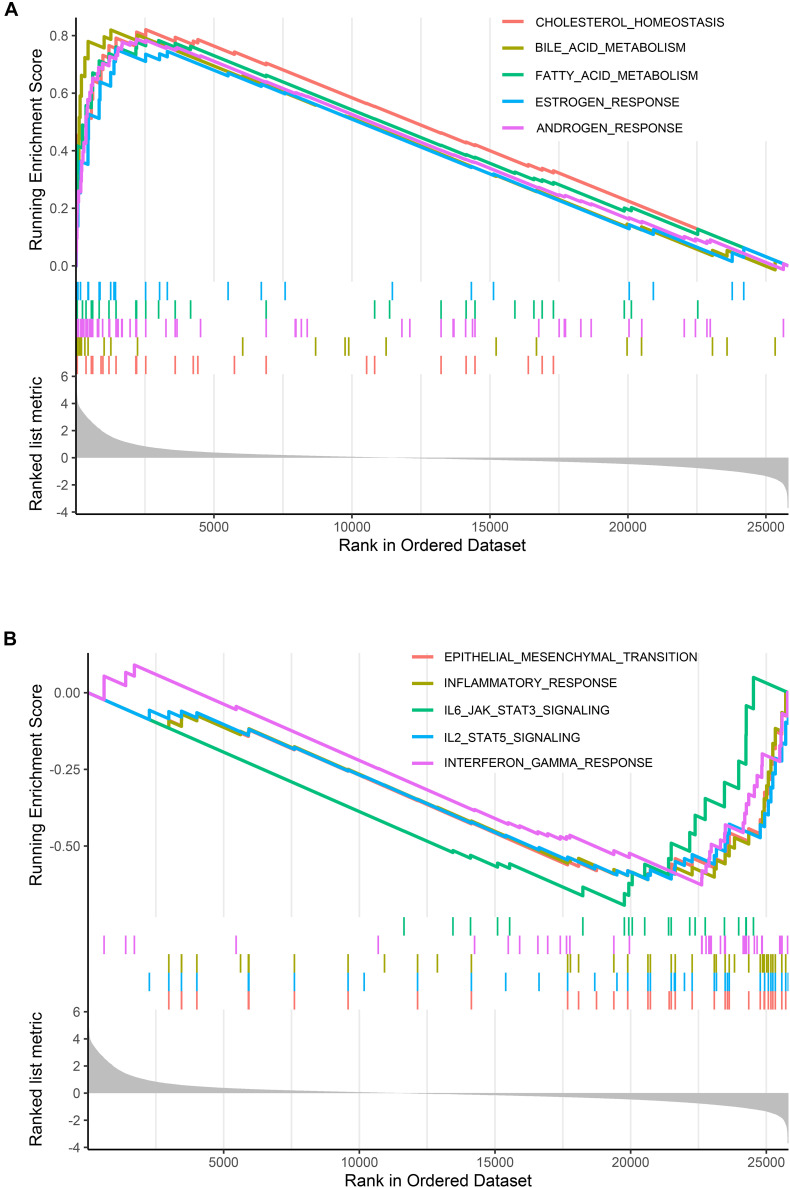
Gene set enrichment analysis (GSEA). **(A)** Top five gene sets enriched in cystic CC group. **(B)** Top five gene sets enriched in fusiform CC group.

The large differences between DEGs observed in cystic and fusiform CCs suggest that these two major subtypes are due to different molecular mechanisms. In order to investigate the relevant MFs and pathways of the DEGs between these two major subtypes, we used GSEA software to compare the pathways between cystic and fusiform CCs. The cystic CC group showed many enriched pathways related to endocrine metabolism and hormone regulation compared with the fusiform CC group (Figure 2A). In contrast, the pathways enriched in the fusiform CC group mainly included those associated with immune response and cell morphology (Figure 2B). These results suggest that cystic and fusiform CCs might be two completely different diseases.

### The Weighted Gene Co-expression Modules of CCs

**FIGURE 3:**
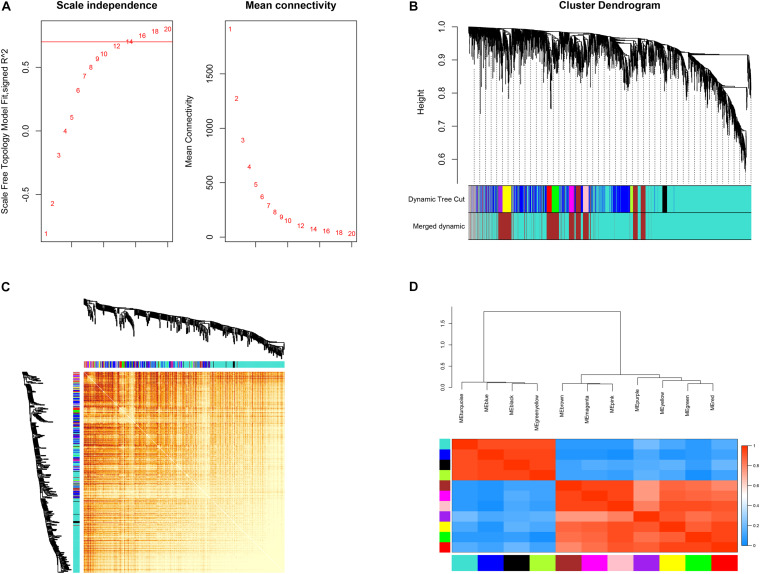
Construction of co-expression modules. **(A)** Determination of soft-threshold power. **(B)** The cluster dendrogram of genes. Genes that could not be clustered into one of these modules were assigned to the gray module. Every gene represents a line in the hierarchical cluster. **(C)** Network heatmap plots of genes selected for weighted gene co-expression network analysis (WGCNA) construction. **(D)** Heatmap plot of the adjacencies in the eigengene network.

Connectivity between genes in the network formed the scale-free network distribution when 0.8 was set as the correlation coefficient threshold, the soft-thresholding power was selected as 14 (Figure 3A). Through WGCNA, total 12 co-expression modules were constructed (Figure 3B). The module comprising most genes was the turquoise one, followed by the blue module. The genes that could not be included in any of the modules were put into the gray module, which was reserved for genes identified as not co-expressed. Moreover, these modules were independent of other modules (Figure 3C). The adjacency heatmap of eigengene showed that modules were divided into two clusters (Figure 3D). One cluster included four modules (turquoise, blue, black, and green–yellow modules), while the other included seven modules (brown, magenta, pink, purple, yellow, green, and red modules). We found that the three pairs of modules had higher adjacencies, and they were the black and green–yellow, magenta and pink, and green and red modules, respectively.

### Gene Co-expression Modules Correlated With Clinicopathological Traits

**FIGURE 4:**
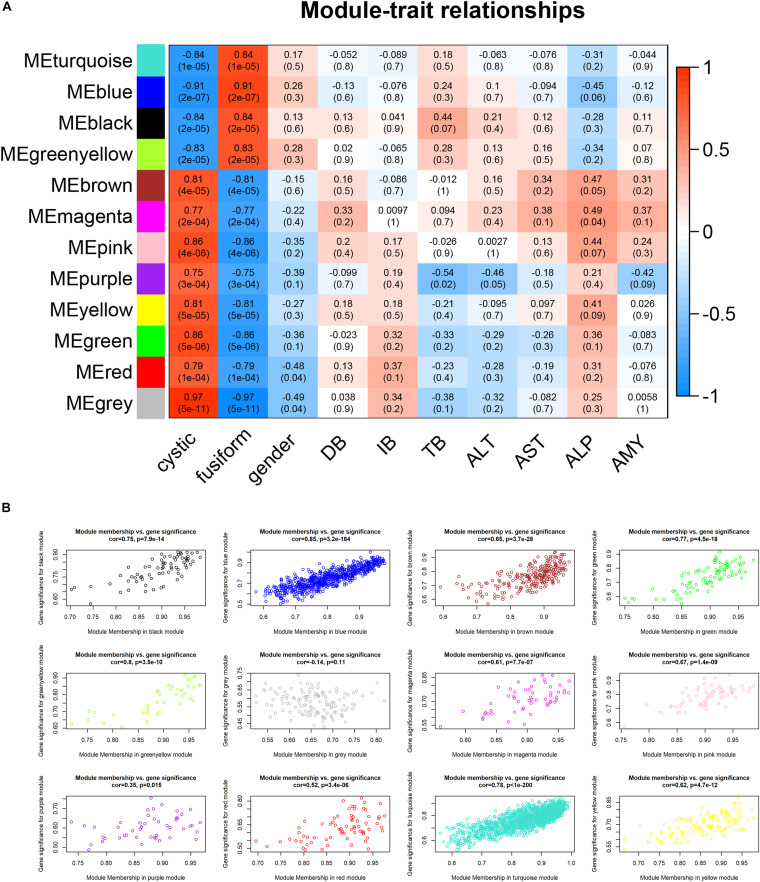
Relationship between modules and clinical traits. **(A)** Heatmap of the module–trait relationships. **(B)** Scatterplots of gene significance (GS) vs. module significance (MS) in modules.

To determine the clinical significance of the modules, we analyzed the correlation between the above modules and clinical parameters. Module–trait correlation analyses showed that multiple modules were related to CCs (Figure 4A). Each module might represent specific clinical traits of CC patients, and highly co-expressed genes in the same module have potential biological significance. The blue module was significantly positively associated with fusiform CCs (*R* = 0.91, *p* = 2e–7), which indicated that the blue modules were most significantly associated with the CC subtype. Therefore, the blue module was treated as CC subtype-related module in subsequent analyses. To ensure the reliability of the results, we performed correlation analysis between GS for CC subtype and MM for genes in each module to test whether MM values were closely correlated with CC subtypes. The results showed that the correlation coefficient between GS for CC subtype and MM was highest in the blue module (Figure 4B); that is, the blue module was the most positively correlated with CC subtype, which was consistent with the above findings.

### Functional Enrichment Analysis Results of Interesting Gene Sets

**FIGURE 5:**
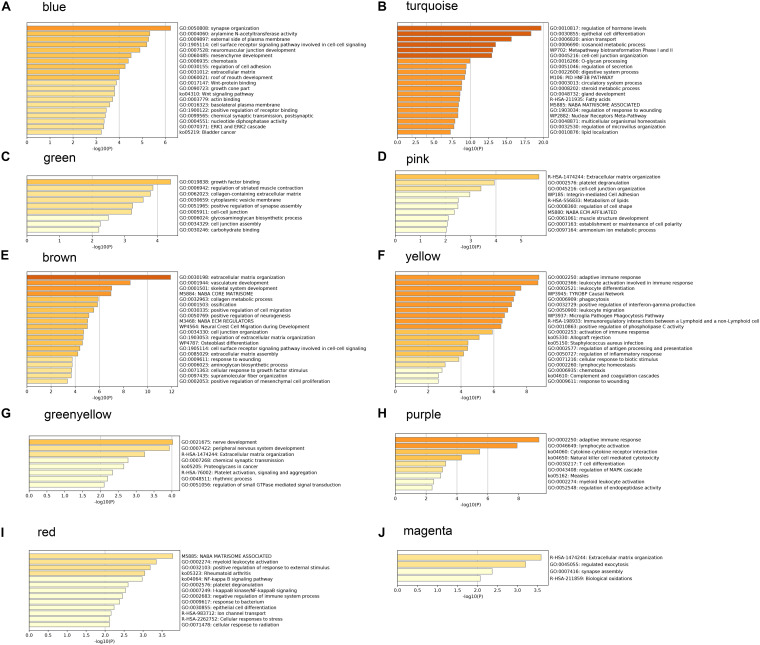
Gene ontology analysis and Kyoto Encyclopedia of Genes and Genomes (KEGG) pathway enrichment results for genes in modules related to histopathological subtypes. **(A)** The enrichment results for genes in blue module. **(B)** The enrichment results for genes in turquoise module. **(C)** The enrichment results for genes in green module. **(D)** The enrichment results for genes in pink module. **(E)** The enrichment results for genes in brown module. **(F)** The enrichment results for genes in yellow module. **(G)** The enrichment results for genes in greenyellow module. **(H)** The enrichment results for genes in purple module. **(I)** The enrichment results for genes in red module. **(J)** The enrichment results for genes in magenta module.

In order to understand the possible biological function of the modules related to histopathological subtypes, enrichment analysis was performed for the genes in each module. The genes in the blue module were mainly enriched in synapse organization, arylamine N-acetyltransferase activity, external side of plasma membrane, and so on (Figure 5A). These results suggest that these genes are closely related to cellular component organization or biogenesis. The functional enrichment analysis in other modules showed similar results (Figures 5B–J).

### Protein–Protein Interaction Network of Selected Modules

**FIGURE 6:**
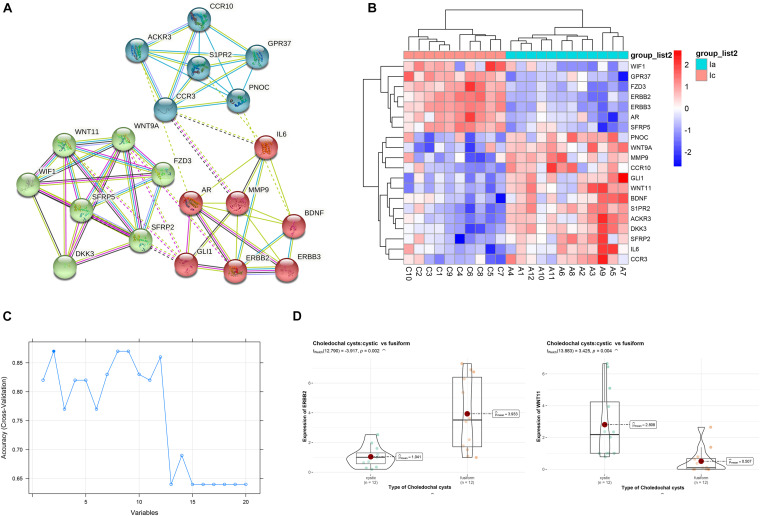
Identification of hub genes. **(A)** The protein–protein interaction (PPI) network. **(B)** Heatmap of the 20 genes. **(C)** Support vector machine recursive feature elimination (SVM-RFE) algorithm to screen hub genes. **(D)** The violin plots of genes between cystic and fusiform CCs.

To further understand the intrinsic linkage of genes in the blue module, we built a PPI network using the STRING database. Based on the criteria that MM > 0.80 and GS > 0.20, a total of 145 DEGs with high connectivity in blue modules were screened as candidate hub genes. Due to the large node network, we mined the submodules using MCODE software in order to more accurately find the genes affecting the subtype of CCs. A total of 20 nodes and 61 edges were obtained (Figure 6A). Enrichment analysis displayed that the genes in the three modules were chiefly concerned with convergent extension involved in axis elongation, C-C chemokine receptor activity, lateral plasma membrane, and Wnt signaling pathway (Table 2). As shown in heatmap (Figure 6B), the 20 candidate hub genes could discriminate between the patients with cystic CCs and the patients with fusiform CCs.

**TABLE 2:** The enrichment analysis of candidate hub genes.

**Term ID**	**Term description**	**Count**	**False discovery rate**	**Matching proteins in network**
hsa04310	Wnt signaling pathway	6	5.29E–07	SFRP5, WNT9A, SFRP2, WIF1, WNT11, FZD3
hsa04060	Cytokine–cytokine receptor interaction	4	0.0012	ACKR3, CCR10, IL6, CCR3
hsa04151	PI3K–Akt signaling pathway	4	0.0025	ERBB3, ERBB2, IL6, BDNF
GO:0060028	Convergent extension involved in axis elongation	2	0.0004	SFRP2, WNT11
GO:0048570	Notochord morphogenesis	2	0.00074	GLI1, WNT11
GO:1900122	Positive regulation of receptor binding	2	0.00087	MMP9, BDNF
GO:0004888	Transmembrane signaling receptor activity	10	4.75E–06	SFRP5, ERBB3, ERBB2, ACKR3, SFRP2, GPR37, CCR10, FZD3, CCR3, S1PR2
GO:0017147	Wnt-protein binding	4	4.75E–06	SFRP5, SFRP2, WIF1, FZD3
GO:0038023	Signaling receptor activity	11	4.75E–06	SFRP5, ERBB3, ERBB2, ACKR3, SFRP2, GPR37, CCR10, AR, FZD3, CCR3, S1PR2
GO:0005576	Extracellular region	11	0.00085	SFRP5, ERBB3, WNT9A, SFRP2, WIF1, PNOC, WNT11, MMP9, DKK3, IL6, BDNF
GO:0005615	Extracellular space	8	0.00085	SFRP5, ERBB3, WNT9A, SFRP2, WNT11, MMP9, DKK3, IL6
GO:0043235	Receptor complex	4	0.0081	ERBB3, ERBB2, GPR37, IL6

*SFRP5, secreted frizzled-related protein 5; WNT9A, protein Wnt-9a; SFRP2, secreted frizzled-related protein 2; WIF1, Wnt inhibitory factor 1; WNT11, protein Wnt-11; FZD3, frizzled-3; ACKR3, atypical chemokine receptor 3; CCR10, C-C chemokine receptor type 10; IL6, interleukin-6; CCR3, C-C chemokine receptor type 3; ERBB3, receptor tyrosine-protein kinase erbB-3; ERBB2, receptor tyrosine-protein kinase erbB-2; BDNF, brain-derived neurotrophic factor; GLI1, zinc finger protein GLI1; MMP9, matrix metalloproteinase-9; GPR37, prosaposin receptor GPR37; S1PR2, sphingosine 1-phosphate receptor 2; AR, androgen receptor; PNOC, prepronociceptin; DKK3, dickkopf-related protein 3.*

### Hub Gene Verification

We then applied the SVM-RFE algorithm to filter the 20 candidate hub genes in order to identify the optimal feature in CC subtype. Finally, ERBB2 and WNT11 (maximal accuracy = 0.87) were identified as the optimal feature genes to construct the classification model using an SVM (Figure 6C). The qRT-PCR experiments on 12 cystic CCs and 12 fusiform CCs demonstrated that the mRNA expression of ERBB2 was significantly lower in cystic CC samples than that in fusiform CC samples (*t* = 3.425, *p* = 0.004), whereas the mRNA expression of WNT11 was significantly higher in cystic CC samples than that in fusiform CC samples (*t* = −3.917, *p* = 0.002) (Figure 6D).

## Discussion

The main characteristic of CC is cystic or fusiform dilatation of extrahepatic bile duct. Treatment of CCs depends upon the type of cyst (Pastor et al., 2021). The clinical characteristics of CCs can be measured; the underlying molecular mechanisms for the clinical subtypes are unknown. Therefore, to better understand and treat CCs, it is pivotal to comprehensively investigate their molecular mechanisms. In this study, we used the DEGs from CC RNA-seq to construct weighted gene co-expression network. Key gene modules associated with the subtype of CC were identified, and the candidate hub genes in modules were detected and their related biological functions and pathways were analyzed. These results have enlightened our knowledge about CCs and might provide some potential molecular therapeutic targets.

We identified 6,463 DEGs between cystic CCs and fusiform CCs. The DEGs were mainly involved in BPs associated with epithelial cell differentiation, regulation of hormone levels, and extracellular matrix. These results indicated that BPs and pathways differed significantly between cystic CCs and fusiform CCs. In addition, GSEA showed that the pathways enriched in cystic CCs are all related to metabolism and hormone regulation, indicating that abnormal bile duct cells might allow them to maintain pathological growth by providing anabolic intermediates for biosynthesis. GSEA indicated that various immune inflammatory responses were strengthened in fusiform CCs compared to cystic CCs, which is consistent with the actual clinical situation. Patients with fusiform CCs usually have severe symptoms such as cholangitis and pancreatitis (Diao et al., 2011). Overall, our enrichment analysis results indicate that BPs and pathways related to inflammatory and metabolism have a critical impact on CCs.

Weighted gene co-expression network analysis can detect clusters of highly correlated genes and presents many unique advantages over other methods and has been widely used in biomedical research (Hu and Bi, 2020). We identified 12 modules and found strong correlations of blue module with CC subtypes. The result suggested that genes within the blue modules may serve as potential markers between cystic and fusiform CCs. To identify key candidate genes in the blue modules, a PPI network was constructed, and genes were filtered with MCODE plug-in. We found that the genes were enriched in Wnt signaling pathway. The key signaling pathways are closely related to the basic developmental processes and the control of asymmetric cell division (Zong et al., 2021). A previous study found that the Wnt pathway was activated in congenital CCs, but the activation level was lower than that in cholangiocarcinoma (Chen et al., 2016). Because CCs belong to a group of diseases characterized by chronic epithelial inflammation and recurrent cholangitis, a carcinoma can occur anywhere along the bile duct. We need to further elucidate the role of the Wnt pathway in CCs. There may be a Wnt inhibitor that prevents malignant transformation in CCs.

We then applied the SVM-RFE algorithm to filter the 20 candidate hub genes in the blue module to identify the optimal feature in CC subtypes. We found that ERBB2 and WNT11 were the optimal feature genes to distinguish cystic and fusiform CCs. The qRT-PCR experiments showed that the mRNA expression of ERBB2 was significantly lower in cystic CCs, whereas the mRNA expression of WNT11 was significantly higher in cystic CCs. ERBB2, also known as HER2, encodes a member of the epidermal growth factor receptor family of receptor tyrosine kinases. The protein encoded by ERBB2 is detected in the surface epithelium in large and septal bile ducts and are not detected in peripheral small ducts (Chow et al., 1995). High expression and activation of ERBB2 have been found in biliary lithiasis and primary sclerosing cholangitis and have been implicated in pathologic proliferation of cholangiocytes (Pellat et al., 2018). Immunohistochemistry of adult biliary cysts revealed variable expression of ERBB2 in cyst epithelia and suggested that cyst formation happens in a non-proliferative way (Waanders et al., 2008). The anti−ERBB2 therapy, pertuzumab, inhibited the *in vivo* growth of subcutaneous tumors in xenografted models (Kawamoto et al., 2015). Many risk factors for cholangiocarcinoma are associated with chronic inflammatory diseases, and the immune system plays a crucial role in the etiology of cholangiocarcinoma. Uncovering the molecular mechanisms of inflammatory response in chronic bile duct disease and the pathways leading to neoplastic transformation of bile duct epithelial cells is a crucial step in developing new strategies to prevent tumor development. In cholangiocarcinoma, ERBB2 is a key mediator of bile duct carcinogenesis, and ERBB2 overexpression and hyperactivation are present throughout tumorigenesis (Jacobi et al., 2021). In our study, ERBB2 expression was found to be increased in the fusiform CCs. We hypothesized that overexpression of ERBB2 may be a risk factor for malignant transformation of fusiform CCs. Evaluation of anti-HER2 agents in fusiform CCs may produce clinical implications. The protein encoded by WNT11 has been implicated in several developmental processes, including regulation of cell fate and patterning during embryogenesis (Monga, 2015). WNT11 is notably upregulated in a liver injury model, and it may be arising from macrophages (Jiang et al., 2019). Clinical and preclinical studies have shown that activation of the Wnt signaling pathway plays a key role in the induction and progression of cholangiocarcinoma (Zhang et al., 2020). In bile duct-ligated mice, adenovirus carrying Dickkopf-1 (WNT antagonist) was able to significantly reduce cholangiocyte proliferation. In addition, WNT antagonists stimulated the secretion of pro-inflammatory mediators by cystic epithelial cells (Cannito et al., 2018). In our study, WNT11 was found to be overexpressed in cystic CCs, suggesting that WNT11 may be associated with abnormal cholangiocyte proliferation. Our results indicate that ERBB2 and WNT11 may play critical roles in CCs. If the results from further studies support our findings, ERBB2 and WNT11 may be promising diagnostic or therapeutic markers for CCs.

This study has some limitations. First, further experiments that will elucidate the molecular mechanism of these candidate hub genes on CCs are needed. Second, like any analysis method, reliable results depend essentially on more high-quality samples. The sample size is relatively small. Therefore, further studies that include more clinical samples will be needed to confirm our findings and to more accurately clarify the possible mechanisms.

Overall, we used WGCNA to identify gene co-expression modules associated with CCs and revealed the candidate hub genes and potential molecular mechanisms related to CC subtypes. As far as we know, this is the first study to investigate the gene expression profile characteristics of patients with CCs. In addition, we identified candidate hub genes and BPs and pathways. These results might be helpful for personalized treatment and are of great clinical significance for CCs.

## Data Availability Statement

The RNA-seq raw data in this article have been deposited in NCBI’s Gene Expression Omnibus and are accessible through GEO Series accession number GSE179075 (https://www.ncbi.nlm.nih.gov/geo/query/acc.cgi?acc=GSE179075).

## Ethics Statement

The studies involving human participants were reviewed and approved by the West China Hospital of Sichuan University Biomedical Research Ethics Committee. Written informed consent to participate in this study was provided by the participants’ legal guardian/next of kin. Written informed consent was obtained from the individual(s), and minor(s)’ legal guardian/next of kin, for the publication of any potentially identifiable images or data included in this article.

## Author Contributions

BX: study concept, and management and coordination responsibility for the research activity planning. JC: study concept, experiment design, data analysis, and critical revision of the manuscript for important intellectual concept. JY: sample acquisition, analysis and interpretation of data for the work, drafting the work and revising it critically for important intellectual content. YLv: study concept, data analysis and drafting of the manuscript, and implementation of the computer code and supporting algorithms. XX: sample collection, conducting a research and investigation process, data analysis, and drafting of the manuscript. LP: performing the experiments and data collection. QW: sample collection, scrub data, and maintain research data. SP and CA: sample and data collection. YLi: critical advice on study design and found support for this project. All authors contributed to the article and approved the submitted version.

## Conflict of Interest

The authors declare that the research was conducted in the absence of any commercial or financial relationships that could be construed as a potential conflict of interest.

## Publisher’s Note

All claims expressed in this article are solely those of the authors and do not necessarily represent those of their affiliated organizations, or those of the publisher, the editors and the reviewers. Any product that may be evaluated in this article, or claim that may be made by its manufacturer, is not guaranteed or endorsed by the publisher.

## Footnotes

1
https://www.ncbi.nlm.nih.gov/geo/query/acc.cgi?acc=GSE179075

2
http://www.metascape.org/gp/index.html#/main/step1

3
https://string-db.org/
