# Corrigendum: Molecular characteristics of choledochal cysts in children: transcriptome sequencing

**DOI:** 10.3389/fgene.2023.1219849

**Published:** 2023-05-26

**Authors:** Yong Lv, Xiaolong Xie, Lihui Pu, Qi Wang, Jiayin Yang, Siyu Pu, Chengbo Ai, Yi Liu, Jing Chen, Bo Xiang

**Affiliations:** ^1^ Laboratory of Pediatric Surgery, Department of Pediatric Surgery, West China Hospital, Sichuan University, Chengdu, China; ^2^ Department of Critical Care Medicine, West China Hospital, Sichuan University, Chengdu, China; ^3^ Liver Transplant Center, Department of General Surgery, West China Hospital, Sichuan University, Chengdu, China; ^4^ Department of Rheumatology and Immunology, Rare Diseases Center, Institute of Immunology and Inflammation, Frontiers Science Center for Disease-Related Molecular Network, West China Hospital, Sichuan University, Chengdu, China

**Keywords:** transcriptome, choledochal cysts, weighted gene co-expression network analysis, children, bioinformatics

In the published article, there was an error in the **Author** list, and Author “Jiayin Yang” was erroneously excluded. The corrected **Author** list appears below.

“Yong Lv, Xiaolong Xie, Lihui Pu, Qi Wang, Jiayin Yang, Siyu Pu, Chengbo Ai, Yi Liu, Jing Chen, Bo Xiang.”

In the published article, there was an error regarding the **Affiliations**. A new affiliation was added for “Jiayin Yang”: “Liver Transplant Center, Department of General Surgery, West China Hospital, Sichuan University, Chengdu, China.”

In the published article, there was an error in the **Author Contributions** as the contributions of Jiayin Yang were erroneously excluded. The correct **Author Contributions** appear below:

“BX: study concept, and management and coordination responsibility for the research activity planning. JC: study concept, experiment design, data analysis, and critical revision of the manuscript for important intellectual concept. JY: sample acquisition, analysis and interpretation of data for the work, drafting the work and revising it critically for important intellectual content. YLv: study concept, data analysis and drafting of the manuscript, and implementation of the computer code and supporting algorithms. XX: sample collection, conducting a research and investigation process, data analysis, and drafting of the manuscript. LP: performing the experiments and data collection. QW: sample collection, scrub data, and maintain research data. SP and CA: sample and data collection. YLi: critical advice on study design and found support for this project. All authors contributed to the article and approved the submitted version.”

The authors apologize for this error and state that this does not change the scientific conclusions of the article in any way. The original article has been updated.

